# Exploring the Effect of a Nature-based Virtual Reality Environment on Stress in Adolescents

**DOI:** 10.3389/frvir.2022.831026

**Published:** 2022-04-08

**Authors:** Elin A. Björling, Jennifer Sonney, Sofia Rodriguez, Nora Carr, Himanshu Zade, Soo Hyun Moon

**Affiliations:** 1 Human Centered Design and Engineering, University of Washington, Seattle, WA, United States; 2 Department of Child, Family, and Population Health Nursing, University of Washington, Seattle, WA, United States

**Keywords:** virtual reality, adolescents, perceived stress, pilot study, mixed-methods

## Abstract

Adolescent mental health is a growing public health issue, with 30% of teens reporting increased stress and 20% of adolescents suffering from depression. Given the scarcity and lack of scalability of mental health services available, the use of self-administered, evidence-based technologies to support adolescent mental health is both timely and imperative. We conducted a mixed-methods pilot study with 31 adolescents ages 14–19 (m = 17.97) to explore the self-administration of a nature-based virtual reality tool. Participant use of the VR environment ranged from 1 to 10 sessions (m = 6.6) at home over a 2-week period while reporting their daily stress and mood levels. All participants completed all of the study protocols, indicating our protocol was feasible and the VR environment engaging. Post-study interviews indicated that most participants found the VR tool to be relaxing and helpful with stress. The themes of Calm Down, Relaxation, and Escape emerged to articulate the participants’ experiences using the VR environment. Additionally, participants provided rich data regarding their preferences and activity in the VR environment as well as its effect on their emotional states. Although the sample size was insufficient to determine the impact on depression, we found a significant reduction in momentary stress as a result of using the VR tool. These preliminary data inform our own virtual reality environment design, but also provide evidence of the potential for self-administered virtual reality as a promising tool to support adolescent mental health.

## 1 INTRODUCTION

Unlike positive stress that promotes healthy growth, negative daily stressors can have negative consequences for adolescents. In a large survey conducted by the American Psychological Association, 27% percent of US teens reported very high levels of daily stress and 31% reported feeling overwhelmed as a result of stress (American Psychological Association, 2014). In addition, a global survey of adolescents in 72 countries found 66% of students reported feeling stressed about academics and 37% of students felt anxiety relating to schoolwork OECD (2016). Increased stress has been shown to cause both mental and physical illness (Maughan et al., 2013) and negatively impact cognitive function, affecting learning (Vogel and Schwabe, 2016).

Prior to the COVID-19 pandemic, an estimated 32% of adolescents had an anxiety disorder while nearly one in three adolescents reported persistent feelings of sadness or hopelessness (NIMH, 2017; CDC, 2019). A large meta-analysis suggests that globally, clinical rates of anxiety and depression have doubled during the pandemic Elliott et al. (2021). The widespread impacts of school closures, social isolation and loneliness during the pandemic are contributing to further increases in adolescent depression and anxiety (Loades et al., 2020; Orben et al., 2020; Hertz and Barrios, 2021). The pandemic has also increased family relationship stressors (Donker et al., 2021) and financial stressors (Wang et al., 2021). For adolescents with existing mental health issues, the pandemic resulted in increased daily stressors (Kudinova et al., 2021).

This “next wave” of widespread mental health impacts as a result of the pandemic highlights the urgent need for accessible mental health services. Despite effective, evidence-based treatments for anxiety and depression, it is estimated that only half of teens will receive mental health services (Lipari et al., 2016), often through services provided by schools. Pandemic-related school closures remove this safety net and further restrict mental health services access for adolescents. Innovative and accessible mental health interventions are needed to address this critical health need.

Technology-based interventions hold promise as scalable and accessible solutions to increase access to mental health care. An array of technologies have been used to deliver mental health interventions, including web, mobile, and more recently, virtual reality (VR) (Hollis et al., 2017; Halldorsson et al., 2021). Consisting of a VR headset that displays simulated environments for exploration or interaction, VR is considered an intuitive and immersive experience. A systematic review found many applications of VR among adults with various mental health conditions, including anxiety, schizophrenia, substance use disorders and eating disorders (Freeman et al., 2017). VR has also been shown to be effective in treating adults with posttraumatic stress disorder (Deng et al., 2019), phobias (Donker et al., 2019), and perceived stress in military personnel (Pallavicini et al., 2016). Among youth, the literature is far more sparse, with the majority of studies focused on VR as an intervention for pain related to procedures and/or burn care (Jeffs et al., 2014; Eijlers et al., 2019). The use of VR as an intervention for adolescent mental health represents an emerging area of inquiry (Schleider et al., 2019; Halldorsson et al., 2021).

Stress is a known precursor to anxiety and depression (Twenge et al., 2019). As such, interventions for stress represent a potential opportunity to prevent more serious sequelae such as anxiety and depression. A recent study exploring the use of VR for a mindfulness-based stress-management intervention among healthcare professionals found that VR was feasible with preliminary efficacy on improving mood (Soh et al., 2021). Use of VR as a stress intervention holds promise as a self-administered, convenient intervention.

### 1.1 Stress and Adolescent Mental Health

The cumulative impact of everyday stressors, particularly chronic stressors, has been shown to have a negative impact on teen mental health (Hamilton et al., 2016). Chronic, daily stress has negative physiological (McEwen, 2017) and psychological (Bolton et al., 2017) outcomes for adolescents, including depression (Maughan et al., 2013) and anxiety (Anyan and Hjemdal, 2016). Chronic stress also negatively impacts cognitive function and learning (Vogel and Schwabe, 2016), both essential for academic performance. Finally, stress as a result of COVID-19 is likely to worsen the mental health of US adolescents (Golberstein et al., 2020) as was found in Chinese adolescents (Chen et al., 2020).

### 1.2 Virtual Reality and Teens

VR is a promising platform for mental health treatment (Bioulac et al., 2018), particularly for adolescents (Cangas et al., 2019). As an intervention tool for adolescents, VR has been shown to treat a range of physical issues such as burn pain (Schmitt et al., 2011), headaches (Shiri et al., 2013), and spinal cord injuries (Flores et al., 2018). On a cognitive level, in a large systematic review, VR has been found moderately successful in improving social development in adolescents with autism spectrum disorders (Mesa-Gresa et al., 2018). VR has also been used to measure and assess attention in both typical and neuroatypical children and adolescents (Diaz-Orueta et al., 2012; Nolin et al., 2016). Exposure therapy in VR has been found effective to reduce anxiety about public speaking in adolescents (Kahlon et al., 2019). Finally, VR has been found feasible and acceptable to promote well-being in adolescents receiving cancer treatments (Tennant et al., 2020).

### 1.3 Nature’s Effect on Mental Health

Access to environmental settings, or *green spaces*, has been shown to improve overall mental health in children and adolescents (Vanaken and Danckaerts, 2018) and significantly reduce problematic behavior in children (Lee et al., 2019). Access to green spaces appears most important for individuals living in urban settings (Nutsford et al., 2013). Shinrin-yoku (forest bathing) is a healing practice in Japan, where people immerse themselves in nature, while mindfully paying attention to their senses (Miyazaki, 2018). In a systematic review, forest bathing was found effective at reducing depression and anxiety in adults (Kotera et al., 2022) and improve well being (Antonelli et al., 2021) in adults. In youth, forest bathing has been shown to reduce perceived anxiety (Guan et al., 2017). Unfortunately, accessing a forest or nature is not always feasible, especially for urban dwelling adolescents.

### 1.4 Preliminary User Research

Given the limited data on adolescent use of virtual reality in adolescents, our team previously explored the usability and engagement of virtual reality for adolescents. In a series of small user studies, we conducted: 1) design sessions with teens exploring relaxation experiences design ideas for VR activities intended to help them to manage stress and 2) usability and feasibility of commercial and home-made VR environments. In total we had 34 adolescents who worked with us in four open design sessions and/or using VR headsets in four public library settings. Adolescents ranged in age from 13 to 19 (M = 18.1), 74.2% were female, and their self-identified ethnicity ranged from Asian (55.8%), Mixed Race (17.6%), White (11.8%), Black or African American (11.8%) to Hispanic (2.94%).

From these user studies we learned the following: 1) teens strongly prefer a nature-based VR environment and found nature environments to be relaxing and engaging. 2) Teens enjoyed being around animals, rather than people, when using VR for relaxation. 3) Almost all teens preferred ambient music along with the sounds of nature during their VR experiences. 4) Active environments were more desirable than passive environments. Teens also enjoyed interacting with objects and making choices about their environment. One particular commercial environment, Nature Treks VR (Greener Games, 2020), was rated as the most relaxing among teens.

Our preliminary user studies provided anecdotal evidence that Nature Treks VR may be a feasible and potentially effective VR environment to reduce adolescent stress. Building upon these findings, the purpose of this project was to conduct a mixed-methods pilot study to explore the effect of Nature Treks VR on perceived stress in three cohorts of adolescents.

Given our data from our preliminary user studies, our current study sought to answer the following questions:

- RQ1: How do teens use a nature-base VR environment in a self-administered setting?
- RQ2: What is the emotional experience of using a nature-base VR environment?
- RQ3: What is the effect of a nature-base VR environment on mood and perceived stress?

## 2 METHODS

This study used a concurrent, mixed-methods design. Quantitative and qualitative data were captured simultaneously throughout the study and culminated in a qualitative exit interview. The University Institutional Review Board approved this study.

### 2.1 Recruitment and Sample

The study used a convenience sample that consisted of three cohorts of teens recruited from spring 2019 through autumn 2020. Recruitment occurred via social media (Facebook, Twitter, LinkedIn), listservs, and word of mouth. For Cohort 1, physical study flyers were posted in the University dormatories as well. Eligibility criteria for all cohorts included: 1) reside in the greater Seattle area, 2) reliable home internet access, and 3) have a phone with SMS capability. Additional eligibility criteria for Cohorts 1 and 2 included: 1) undergraduate student at the University of Washington (blinded for review) and 2) aged 18 or 19 years. Additional cohort 3 eligibility included: 1) ages 14 years or older and 2) middle school or high school student. Exclusion criteria included conditions that would preclude the use of VR including visual or hearing impairments, severe developmental delay, or a history of vertigo, epilepsy, or extreme motion sickness. To maximize the likelihood of our cohorts experiencing stress during the study, cohorts were intentionally recruited during the final weeks of academic terms and/or end of the school year.

### 2.2 Virtual Reality Headset

The Oculus Go https://www.oculus.com/go/ is a stand alone virtual reality headset that comes with a single controller and charging cable. The headset is lightweight, weighing just over one pound, and easy to use as a result of its cordless nature. The headset has built-in spatial audio drivers, providing an immersive experience. It is lined with fabric covered foam to add comfort. It is adjustable through hook and loop fasteners on elastic straps, allowing subjects to find the most comfortable fit. The single three-degrees-of-freedom controller is composed of a clickable touch pad that can be used to move around intuitively, as well as two simple Back and Home buttons, limiting user error. It also has a front trigger that fits ergonomically into the hand and a wrist strap for safety. For the study, other applications were uninstalled when possible to deter participants from using the device for other purposes. In addition, the wireless connection was turned off to prevent participants from connecting their headset to their phone or the internet.

### 2.3 Virtual Environment: Nature Treks Virtual Reality

Nature Treks VR designed by Greener Games https://www.greenergames.net/nature-treks is a commercially available application with color-themed nature environments. Unlike typical VR games, there is no objective or intended activity. Instead, Nature Treks VR is designed to provide an immersive experience that promotes user exploration of the environments. Each environment includes soothing audio and a variety of wildlife. In addition the user can choose the time of day (day or night) and precipitation (rain or snow).

### 2.4 Intake and Exit Measures

#### 2.4.1 Demographics

Participant characteristics were collected in an investigator-developed survey with items that included grade level, gender, race, and ethnicity. Note, gender, race and ethnic identities all included free text response options. An additional item asked the participant to indicate their experience level (never, once, 2–5 times, 6 + times) with various VR technologies, including cardboard, standalone headsets and full headset.

#### 2.4.2 Perceived Stress Scale

The Perceived Stress Scale (PSS) is a 10-item survey that uses a 5-point scaled response (0 = never, 4 = very often) to measure perceived stress during the past month, with higher scores indicating higher stress (Cohen et al., 1994). Items 4, 5, 7 and 8 are reverse scored, then all scale items are summed for an overall score. The PSS has established reliability and validity for adolescents.

#### 2.4.3 Patient Health Questionnaire

The Patient Health Questionnaire (PHQ-9) is a 9-item survey that uses a 4-point scaled response (0 = not at all, 3 = nearly every day) to assess for depression (Kroenke et al., 2001). Scale items are summed for an overall score. PHQ-9 total score established cut-points include minimal depression (1–4), mild depression (5–9), moderate depression (10–14), moderately severe depression (15–19), and 20–27 (severe depression). The PHQ-9 has established reliability and validity.

#### 2.4.4 Cognitive Fusion Questionnaire

The Cognitive Fusion Questionnaire (CFQ-7) is a 7-item survey that uses a 7-point scaled response (1 = never true, 7 = always true) to measure cognitive fusion (Gillanders et al., 2014). Cognitive fusion is a term rooted in cognitive behavioral therapy that reflects the tendency for individuals to act on thoughts as if they are true, which can overwhelm or dominate behavioral regulation. The opposite of cognitive fusion is cognitive defusion, when one is able to distance themselves from their thoughts which, in turn, prevents thoughts from controlling actions. All item responses are summed for an overall score, with higher scores indicating higher cognitive fusion. The CFQ-7 has established reliability and validity.

#### 2.4.5 System Usability Scale

The System Usability Scale (SUS) is a 10-item survey that uses a 5-point scaled response (1 = strongly disagree, 5 = strongly agree) to measure the usability of a system (Bangor et al. (2008)). Scoring consists of several steps: 1) sum all odd numbered items (1,3,5,7,9) and subtract 5, 2) sum all even numbered items (2,4,6,8,10) and subtract that total from 25, 3) sum the totals from steps 1 and 2, and 4) multiply the total from step 3 by 2.5. Total scores range from 0 to 100; a score of 68 or higher is considered above average usability. The SUS has widespread use as well as established reliability and validity. At the end of the study, participants were asked to rate their experience of using the VR system at home as a whole using this scale.

### 2.5 Within-Day Stress and Mood Surveys

Given the tendency for mood and stress to fluctuate throughout the day, we employed a brief 2-item ecological momentary assessment to measure subject mood and stress periodically throughout the study. A visual analog scale (VAS) ranging from 0 to 100 (0 = no stress at all/poor mood, 100 = most stress experienced/great mood) was used for each item. VASs are commonly used to reliably measure momentary perceived stress, even over very brief time intervals (Lesage et al. (2012); Cella and Perry (1986)). These surveys were captured from participants in cohorts 2 and 3 by text three times per day (morning, mid-day, and evening) during the 2-week study period.

### 2.6 VR Session Log

**FIGURE 1 |:**
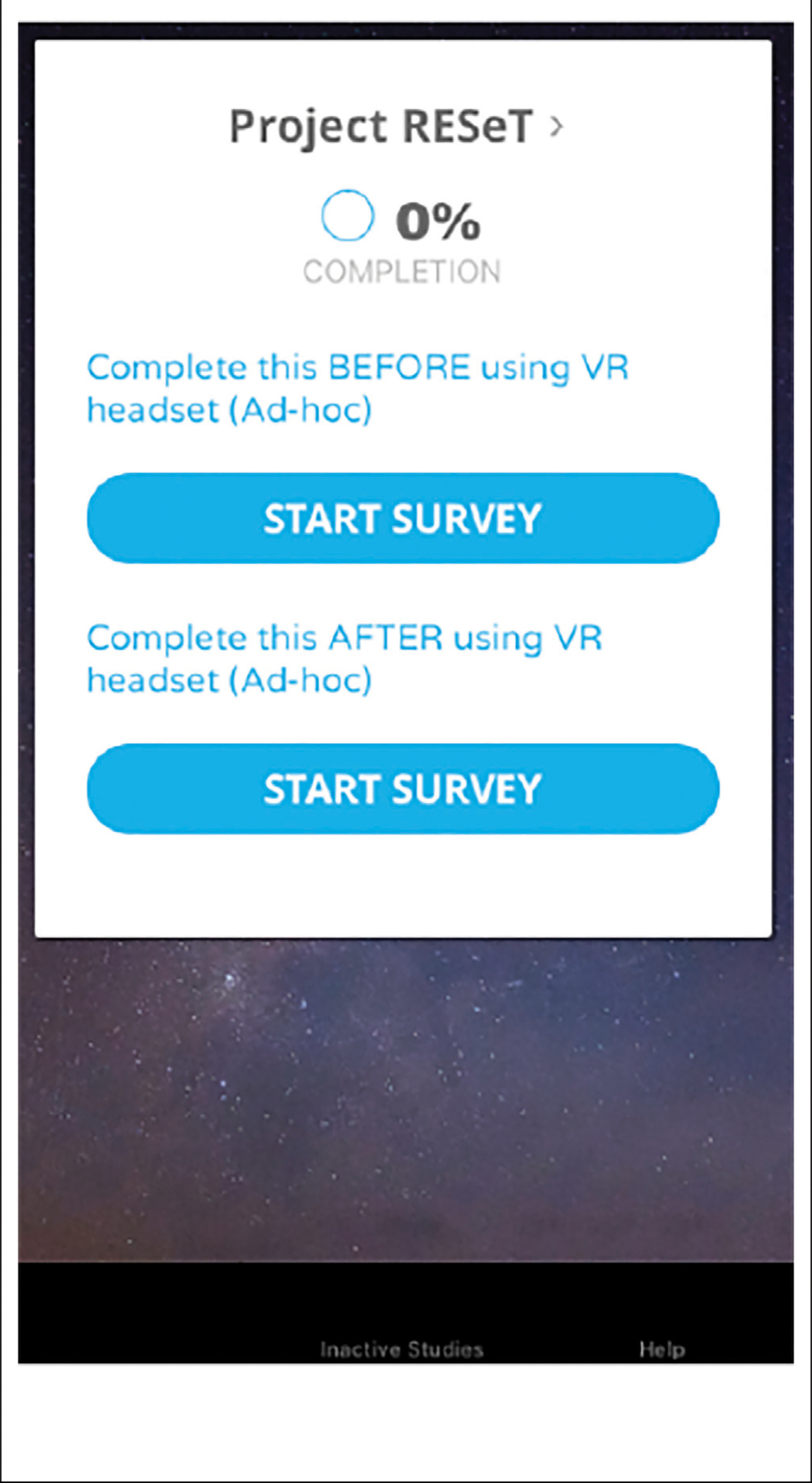
Welcome screen from the participant initiated pre and post Virtual Reality log application.

The investigator-developed VR session log was used to track VR session use date and duration, mood and stress ratings before and after each use, information about the VR environments visited, and participant comments. For consistency, pre- and post-session mood and stress were assessed using the momentary stress and mood VAS scales described earlier. Figure 1.

### 2.7 Semi-Structured Exit Interview

An investigator-developed semi-structured interview was completed at the end of the study to examine overall study usability, solicit participant feedback about the study, protocol and environment, and to provide the opportunity for suggested improvements. Seven interview questions were used to guide the interview, such as “Overall, what was your experience in Nature Treks VR?” and “What would you change about the study?“. Interviews lasted approximately 15–20 min and were either live or recorded via videoconferencing software.

### 2.8 Study Procedure

The study sample was comprised of cohorts 1, 2 and 3. Given the novelty of using VR as a self-administered health tool, we iteratively refined our study procedures after each cohort based upon subject and study team feedback. Additionally, cohorts 2 and 3 occurred during the COVID-19 pandemic, which required additional modifications to transition to a remote study protocol described in Sonney et al. (2021). See 2 for an illustration of the study measurement procedures.

#### 2.8.1 Cohort 1

**FIGURE 2 |:**
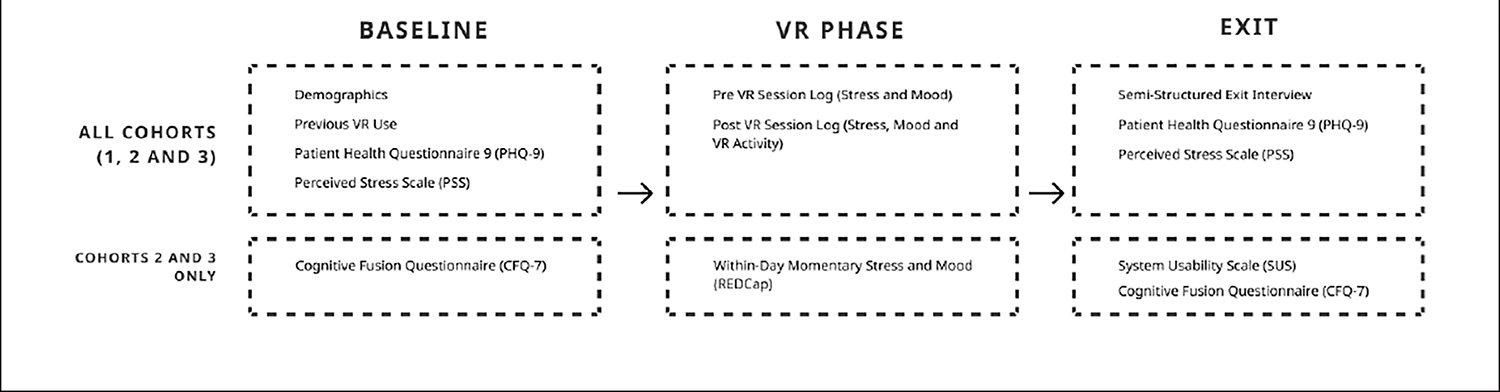
Study measurement and procedures.

Prospective subjects completed an online eligibility screening survey and provided their name and contact information. Study personnel contacted the prospective subject to discuss the project, review the activities, and schedule the subject for an in-person baseline session. At the in-person baseline session, following written informed consent, subjects completed a series of digital surveys (Figure 2). Next, subjects were oriented to VR Study Kit, consisting of an Oculus Go (Oculus, Menlo Park, CA) VR headset preloaded with Nature Treks VR, controller, charging cable, and spare batteries. Study personnel showed the subjects how to use the VR headset, access the Nature Treks VR environments, and reviewed a printed on-boarding manual that outlined step-by-step instructions for using the headset. Additionally, subjects were provided a printed instrument booklet containing VAS measures of perceived stress and mood and the VR session log.

Participants were instructed to use the VR headset at least 3 times a week for 2 weeks. For each session, first they were asked to complete the paper mood and stress VAS scales, next use the VR headset for 5–15 min, and then complete the paper mood and stress VAS scales after use. They were also asked to complete the VR session log detailing the date, duration of use, environments visited, and any additional comments. The study team conducted a check-in phone or video conference after 1 week to answer any questions, troubleshoot if necessary, and schedule the in-person exit session. At the end of the 2 week study, participants attended the exit session at which time they returned the VR Study Kit, completed the post-study digital surveys, and participated in a brief exit interview.

#### 2.8.2 Cohorts 2 and 3

Cohort 2 occurred during March 2020 and Cohort 3 in autumn 2020, amidst the COVID-19 pandemic and stay-at-home orders, which necessitated a remote study protocol. REDCap, a secure browser-based data capture and management system, was used for all participant data collection with the exception of the semi-structured interview. Prospective subjects completed the online screening survey, which automatically determined study eligibility. For those who were eligible, REDCap automatically generated the study consent form for review using the “autoarchiver + e-Consent framework”. Participants completed the e-consent procedures and were provided an emailed PDF copy of their signed consent. Note, the parents of subjects under age 18 years were emailed a parent informational letter and the contact information for the study PIs. Following consent, subjects then completed the baseline survey queue (Figure 2). Upon completion of consent and baseline surveys, study personnel contacted the subject to schedule a VR Study Kit delivery to participant homes. Sanitized VR Study Kit deliveries were “no contact”, with study personnel maintaining appropriate distancing, delivering the kit outdoors, and ensuring by visual or text confirmation that the kit was received (Sonney et al., 2021).

Similar to Cohort 1, subjects were advised to use the VR headset at least 3 times per week for 5–15 min. The study team included a VR headset visor with a printed QR code. Subjects were instructed to scan the code prior to VR headset use, which linked to the REDCap momentary stress and mood VAS. After the VR session, subjects completed another set of stress and mood VAS as well as the VR session log. In order to measure stress and mood variability throughout the day, REDCap sent SMS links to the stress and mood VAS for completion in the morning, noon, and evening. The study team reached out via text message to schedule a 24-h check-in, a 1 week check-in and a final semi-structured interview. Subjects were also told via text message that they could text if any issues arose. The study team conducted the 1 week check-in, similar to Cohort 1, *via* phone or videoconference. Post-study surveys were sent *via* REDCap and the semi-structured interview occurred *via* videoconference. After study completion, the study team retrieved the VR study kit and completed a sanitation procedure.

#### 2.8.3 VR Session Protocol

**FIGURE 3 |:**
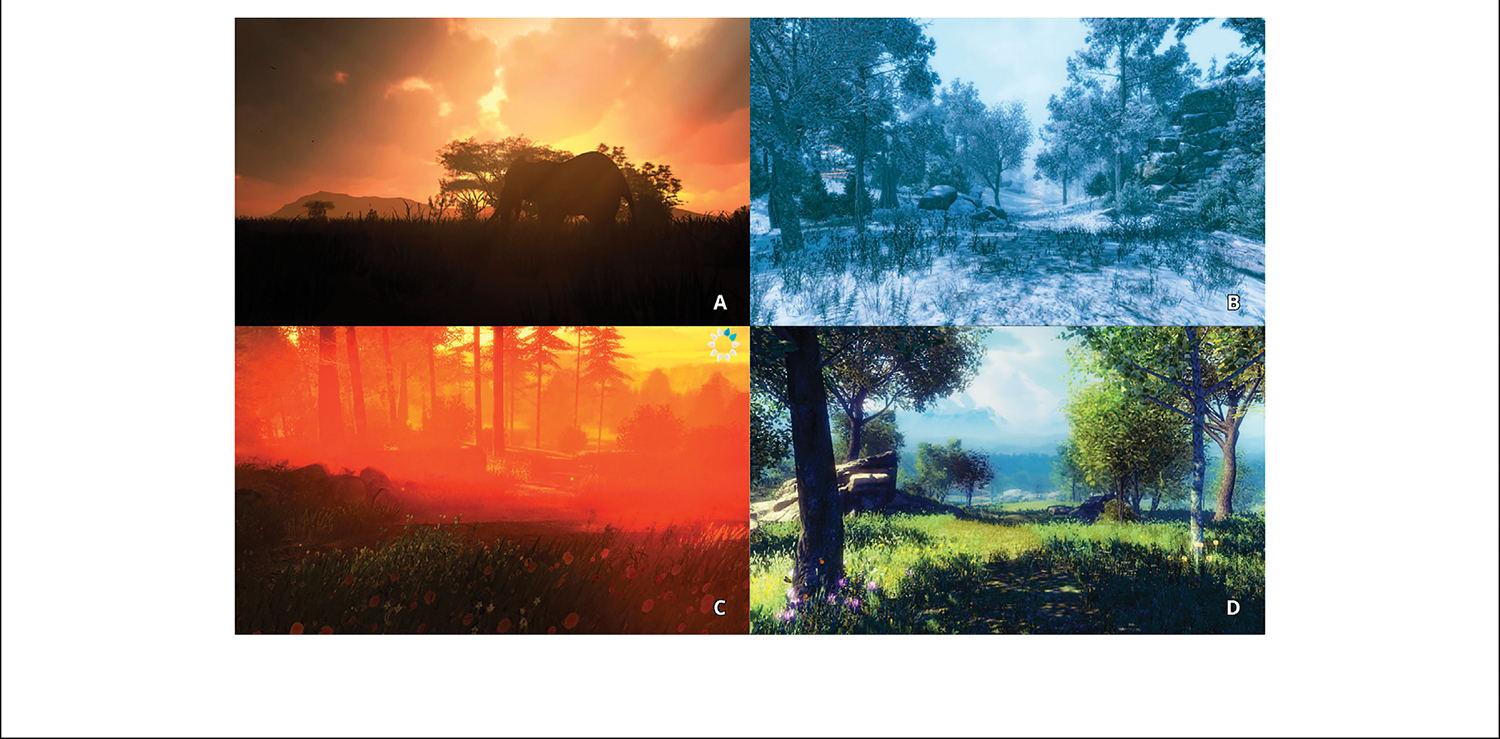
Illustration of the four nature treks VR environments used in the Study. (**A**) Orange Sunset, (**B**) White Winter, (**C**) Red Savanna, and (**D**) Green Meadows. Reproduced with permission from Greener Games.

Following completion of the pre-VR mood and stress VAS scales, participants were instructed to use the headset at least three times a week at any time of their choosing. With the headset on, participants were instructed to navigate to the Nature Treks application and launch it. Participants were then instructed to choose from four of the Nature Treks VR environments: (A) Orange Sunset, (B) White Winter, (C) Red Savanna, and (D) Green Meadows. Figure 3 for an illustration. An underwater, and outer space environment were available in the application, but participants were asked not to use these environments. Our preliminary design sessions with teens found that many teens were startled, over-stimulated, or uncomfortable in these environments.

Participants were also given instructions on how to move around in the environment using both the controller trigger and touch surface. Once finished with the headset for the day, subjects were instructed to exit Nature Treks, remove the headset, and plug it in to charge. Finally, they were prompted to complete a post-VR session log.

### 2.9 Analysis

#### 2.9.1 Determination of a VR Session

Participants initiated the pre and post VR session logs. Therefore, several logs were incomplete due to missing post-session log items. In order to best capture the frequency of VR use, session logs that were initiated, but not completed, were included in VR session frequency, but not duration analyses. For session duration, we only considered sessions that were up to 120 min long to ascertain active participant involvement throughout the session. All incomplete logs were excluded from statistical analyses exploring the effect of VR on stress and mood.

#### 2.9.2 Usage and Usability

Usage metrics (frequency, duration, choice of environment) were analyzed descriptively in R Core Team (2014). The System Usability Scale was also scored and calculated (Bangor et al., 2008).

#### 2.9.3 Qualitative Data

Qualitative data were analyzed using a thematic analysis (Clarke et al., 2015). As part of our mixed-method format, the qualitative data were gathered concurrently with our quantitative instruments and then explored to help contextualize our quantitative findings. Three members of the research team explored the qualitative data from session logs, direct contact with participants, and exit interviews. Analysis began with the extraction of excerpts that felt salient in relation to our research questions around activity in VR, emotional experience, and the effect of VR on stress. From a review of extracted excerpts, open coding was used to create a categorical code book. With the code book, researchers then revisited the data to further contextualize the categorical codes in an effort to represent the depth and breadth of experiences described by participants. This process was repeated until the research team felt we had sufficient evidence to contextualize our study findings.

#### 2.9.4 Effect of Virtual Reality

We conducted paired-samples t-tests to compare the baseline and exit measures of *depression, perceived stress* and *cognitive Fusion*. We also descriptively explored the average reported mood and stress (from within-day surveys) over time for the whole group to explore any patterns.

To explore how self-reported momentary stress and mood changed as a result of using the VR environment, we conducted two separate linear mixed effect regression analyses predicting stress or mood including random effects for participant to control for varying number of sessions per participant. We restricted analyses to only include the momentary time points immediate to and immediate post every VR session and categorized these as “pre” and “post” VR time points. These were included as a dummy coded categorical variable with pre as the reference variable using full maximum likelihood. Finally we conducted a mixed-effects linear regression to predict the change in momentary stress and mood based upon length of the VR session.

## 3 FINDINGS

### 3.1 Study Enrollment and Demographics

Forty-eight individuals completed study screening, 40 were eligible, and 30 enrolled. Reasons for ineligibility included four who lived outside of the study geographic area and four who were not within the age parameters. All 30 participants that enrolled completed all study procedures (we had no attrition). The mean age of participants was 17.97 (SD = 1.37) and 57% were female. Table 1 for more detail on frequency and duration of VR sessions.

**FIGURE 4 |:**
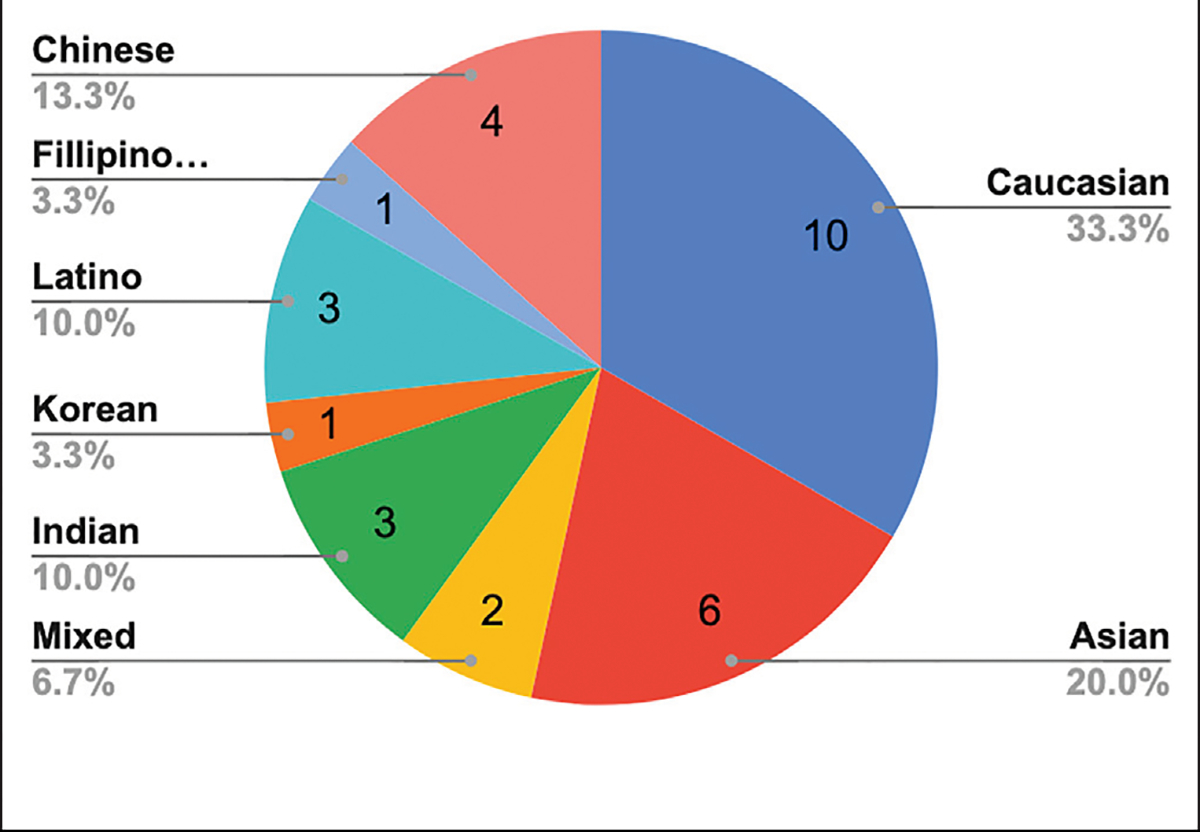
Self-reported ethnicity of the study participants.

Participants self-reported diverse ethnic backgrounds. Figure 4 for more detail. With respect to VR experience, 16.6% (*n* = 5) had never used any type of VR, 56.6% (*n* = 17) had used some type of VR at least once and 20% (*n* = 6) had used some type of VR more than 6 times prior to the study.

### 3.2 RQ1: How do Teens Use VR in a Self-Administered Setting?

Given the minimal published data on self-administered VR, especially in adolescents, we were interested to explore how teens used Nature Treks VR in the real-world, in their daily lives.

#### 3.2.1 Usage and Usability

Usability was measured in cohorts 2 and 3. The mean usability score for the VR system was 77.27 (SD = 3.24) and 76.75 (SD = 4.45) respectively. Typically a system usability score above 70 is considered good and above 80 very good (Bangor et al., 2008), therefore it was clear that participants found the VR system easy to use and accessible. In a stress-reducing VR biofeedback game for adults, the mean SUS score was 80.5 Maarsingh et al. (2019).

Participant VR session logs showed an average of six VR sessions (min = 1 and max = 10) with the headset over the 2-week study period. Table 2 for more detail. Two participants only logged one VR session during the study period and are discussed in more detail below. Males in the study used the VR slightly more often than females (m = 5.85/m = 5.06). Participants typically spent between 15 and 20 min in the headset, and a few reported being in the headset for up to an hour. One participant reported accidentally falling asleep in the headset and recorded a 5-h session that was removed from the descriptive data and efficacy analysis as an outlier.

#### 3.2.2 Disengagement

All participants in our study engaged with the VR device and the Nature Treks environment. Most participants found using the headset comfortable, however, a four participants reported mild symptoms of dizziness, nausea or headache especially after using the headset for an extended period of time.

Three participants told us in the exit interview that they would not continue using the headset if given the opportunity. For one participant, this was because, “After a little while in each environment, things started to feel a little bit claustrophobic.” He also shared, “ (Nature Treks) was not that interesting and there were better VR games out there” (P304, Male, 19 years, VR Session Log). A second participant felt that she had other more effective ways to reduce her stress like exercise and sports.

I think it definitely is calming. It is like quiet natural noises and stuff, which I think has been nice. It is like, not something I would go to for stress relief . . . Like the Nature Treks totally got the outside part but the physical part is something that I also really need (for stress relief). (P312, Female, 19 years, Exit Interview).

A third participant said she would not continue to use the headset because of pain. After using the headset one time, she did not use it again.

Okay, so first of all, it hurts after a while. Like headache, woozy? and just not a good feeling . . . So it is not just me. It hurts. I was just like, wow that’s pain, I want to have fun. (P101, Female, 19 years, Exit Interview).

#### 3.2.3 Activity in Nature Treks

In exploring our exit interviews and VR session participant logs, we saw some common themes that are described in more detail below. Of the four Nature Treks environments (Figure 3, the White Winter environment most popular. Out of 152 environments visited, 52 were to White Winter. White Winter was also identified explicitly as the “favorite” environment for nine (29%) of our participants. Only 15% of participants explored the Orange Sunset environment. Teens generally appreciated the ability to customize the application including the ability to change the time of day and the option to choose different weather settings.

In their exit interviews, the themes of *Winter Nights* and *Lying Down* quickly emerged as many teens described how they used Nature Treks VR.

Many teens described the Winter environment as their favorite environment and how using the night option was also preferred. One teen wrote in their session log, “I really like the Winter environment when it is snowing and nighttime. It is relaxing to just look up at the sky”. (P401, Female, 15 years, VR Session Log). Another teen wrote “Laying down and having snow fall on UR face and watch the sky is very peaceful” (P303, Female, 19 years, VR Session Log).

The night mode option in Nature Trek VR was described as “calm, “peaceful,” and “quiet.” As one teen articulated,

I prefer night mode. Especially for the White Winter . . . And it makes you calm down because it’s dark. And you can just hear the birds or hear whatever other animals are talking. It make you calm down and relieves stress. (P109, Female, 19 years, Exit Interview)

Another common theme in our data describing their activity in VR was, *Lying Down*. Many teens found that to physically lie down during the VR sessions was very relaxing. As one teen stated,

I’ve always had this like fantasy of just like lying underneath a tree and just staring up into the sky for hours. But that’s impossible for my current life because one, the sky isn’t very attractive or two, the time just doesn’t align for that . . . So to be able to do essentially that for 10 mins was very very peaceful. I thought that really nice actually. It’s really comforting. (P102, Male, 18 years, Exit Interview)

I would lay down on my bed with my thing on, and I would turn it into nighttime and I would have little fireflies light up the sky along with the stars, and that was the most serene. I just loved that. It was really calming, really relaxing at the time. (P104, Female, 19 years, Exit Interview)

### 3.3 RQ2: What is the Emotional Experience of Using Nature Treks VR?

As participants described their emotional experiences of using the Nature Treks VR application, the themes of *Calm Down, Relaxation* and *Escape* emerged and are described below.

In our exit interviews, most participants reported overall positive experiences with the application and many reported positive changes in their mood and stress levels that they attributed to the VR experience.

The theme of *Calm Down* was quite prominent in the teens’ data. This theme encompassed the many examples of how they articulated the effect of the VR environment on their emotional state. Quite a few teens described being upset as a result of school, the pandemic, or even the 2020 election. They intentionally used the VR application to help to shift their mood. For example, one teen even suggested that the environment helped her to reflect on her situation.

Before this I was hit with a huge problem that stressed me out a lot. This game calmed me down significantly. While finishing tests last week were something that had to get done, this situation was something I couldn’t do anything about, so the virtual environments were a very good meditation that allowed me to calm down and realize that. I laid down in bed for this one and made it night time and just looked up for most of it. (P310, Male, 19 years, VR Session Log)

Another teen mentioned that they intentionally used the headset in response to negative feelings like discouragement.

A lot of times I did it (used the headset) when I was really discouraged . . . I just put on the headgear and I was like I just don’t want to think about it for a sec . . . I went into the Winter one at nighttime and I was like I just really enjoy the light and I was really enjoying like just the comfy atmosphere of no people. I know nobody gonna pop up. I know I can kinda just be alone in space. That’s like relaxing so . . . I liked that, you can’t just look down at your phone, I turned my phone on silent, I always made sure I was in a place where I wouldn’t be interrupted, and that made it so nice. (P108, Female, 19 years, Exit Interview)

Another teen described Nature Treks as helpful in managing the feeling of being overwhelmed.

Um, I noticed that it definitely like calmed me down a lot. And like made me think about other things like there were a few times where I was just like, had a lot of like overwhelming thoughts. And so, and then when I did it, it like kind of helped to calm those down and like because it’s like the environment is all around you. It’s kind of like harder to be thinking about anything else. (P410, Female, 16 years, Exit Interview)

These descriptions suggested that some teens felt the environment may have helped them the self-regulate and process their emotions by allowing them to focus on the Nature Treks environment.

The emergent theme of *Relaxation* was found in many exit interviews as teens attempted to articulate their experience and the potential value of the VR application. One teen described that the VR environment gave them space to sort out their thoughts and that was relaxing.

I feel like it gives you time to like think a little you know you can like kind of not only is it like relaxing, just to be there, but you kind of get to sort out your thoughts, a little and like work out anything you’re like stressing you out or just not really thinking about a lot. (P406, Male, 17 years, Exit Interview)

Another teen suggested that the relaxation effect was lasting beyond the use of the headset.

I think I felt relaxed for a while after that (after using the headset). . .so I was just relaxed for the rest of the night just because I don’t have anything else to do or to worry about. I feel, I don’t know, I couldn’t really say you know because I don’t really have any stresses than I was on cuz I didn’t use it while I was extremely stressed and trying to get things done. (P103, Female, 19 years, Exit Interview)

The theme of *Escape* was not surprising given the immersiveness of virtual reality. As one teen shared in their VR session log, “I definitely needed the break from homework right now” (P407, Non-binary, 15 years, VR Session Log). A couple of participants also described it as a forced escape specifically from their mobile phone, “I definitely did feel as if it was a good break, especially since I couldn’t really use my phone or anything while I was using it” (P310, Male, 19 years, Exit Interview). Another participant articulated the benefit of being uninterrupted in their experience.

Yeah. I really you know, it just gives you a second to step into a fake world which I think in its own way has a lot of benefits because you don’t need to worry about being interrupted by some person, you don’t have to worry about, you know cars, urban developments. You just kinda like in this landscape that at one point exists in some form and I think that in itself is relaxing. (P108, Female, 19 years, Exit Interview)

#### 3.3.1 Undesirable Interactions

A final category that emerged was *Undesirable Interactions*. Although these represent a small minority of participants, they are important to note to inform future research into the therapeutic effect of VR by reducing stress and improving mood. Participants mentioned only a few things they disliked, one being the large animals that are present in certain environments, such as Orange Sunset. One teen even described being startled by animal sounds, “all of a sudden I’d hear just like a deer make a noise and it would like make me jump in my chair” (P102, Male, 18 years, Exit Interview). Another teen described almost a fear of the wild animals.

I felt really scared, so I just immediately left the environment, to never go there again, um, cuz I know I just felt really scared after that because . . . there was like a cheetah, I think. And now somehow like gave me like a instinct to like stay away from it. (P402, Female, 17 years, Exit Interview)

Encountering the “fakeness” of the environment, such as animals walking through you or through objects, in addition to the forced boundaries were described as “disappointing” or “discouraging” by some teens. As one teen articulated their thought process upon running into a boundary,

It was kinda like “oh, that’s right this is fake”. . .it kinda felt like aww, I want to keep walking into something . . . but the boundaries did also remind you that it’s not real. So that’s the only downside I would say. So it’s kinda like detracted from the experience in the sense that you feel like, oh there is a wall there but you want to keep going because the moose is on the other side of the wall. You know there is a moose right there . . . “I wanna follow you, take me somewhere” (to the moose) (P108, Female, 19 years, Exit Interview)

### 3.4 RQ3: What is the Effect of Nature Treks VR on Perceived Stress and Mood?

It is worth noting that our participants, although self-selected, were likely a stressed sample due to the fact that cohorts 2 and 3 were using Nature Treks VR during the COVID-19 pandemic. The mean PSS score for the sample was 22.83 (SD = 6.97) with most participants scoring in the moderate stress range. The mean stress of our sample was only slightly higher than a 2019 large United Kingdom university sample (*n* = 524) which reported a mean PSS of 19.79 (SD = 6.37) (Denovan et al., 2019).

We found significant changes in momentary stress and mood resulting from the VR sessions. Participant-reported average momentary stress before the use of VR was 52.60 and this average significantly decreased 15.49 points to 36.5 after the session. Similarly we saw momentary mood before the VR session was 49.78 and increased 10.65 point to 60.43 post VR. Table 3 for more detail. We did not find any significant effect of session duration on participants’ momentary stress or mood recorded in VR session logs. (Table 4 for more detail.

**FIGURE 5 |:**
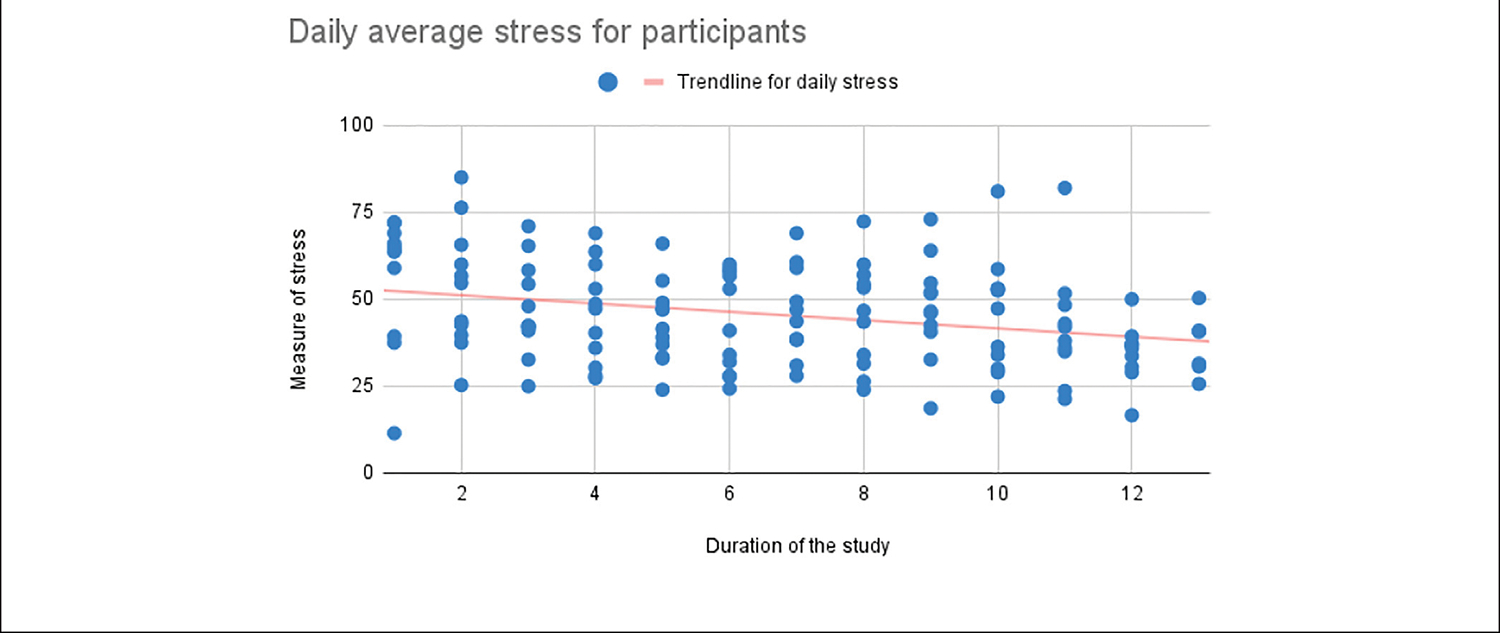
Daily average stress self-reported by the study participants.

**FIGURE 6 |:**
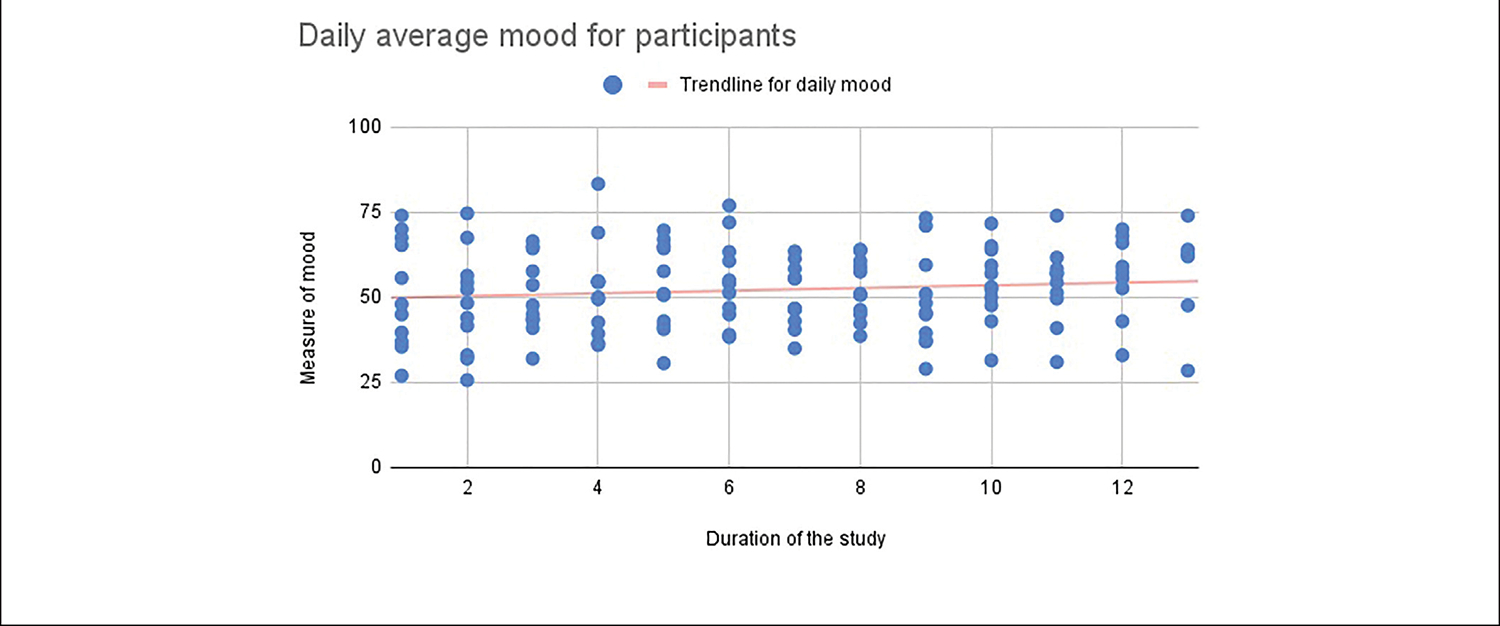
Daily average mood self-reported by the study participants.

Figures 5, 6 illustrate the general decreasing trend of daily average stress and increasing trend of daily average mood as captured in the within-day surveys over the duration of the study.

Similar to quantitative findings, the qualitative data supported participants’ experience of momentary reductions in stress as a result of using the VR environment as evidenced by their exit interviews. Some felt the effect was lasting, “I definitely noticed I felt like a lot more like, a lot less stressed afterwards” (P310, Male, 19 years, Exit Interview). In contrast, several other teens suggested that the stress reducing effect of Nature Treks VR was fleeting. For example,

Overall for me, they really helped me lower stress, but it was good it boosted my feelings and increase my mood. But didn’t decrease my stress as much as I thought. So like when I go in side, when I see all this sites and everything, I feel really happy but, at the end of the day, it didn’t really lower my stress levels, because I still get really stressed after the VR experience . . . During VR, I didn’t really think about anything. I guess it helped me to forget about everything. (P405, Female, 17 years, Exit Interview)

However, the lasting effect of Nature Treks on individual stress levels also may be difficult to perceive and report as illustrated by this participant.

I’m not sure about like long term, I’m not sure if, if like a few hours later, I was really much less stressed. But while I was there, it was a really good like momentary, uh, way to calm down. “(P409, Male, 16 years, Exit Interview)

### 3.5 Other Effects

Outside of our momentary measures capturing changes in self-reported stress over time, we also wanted to measure depression given the strong relationship between chronic stress and depression (Thapar et al., 2012). Our convenience sample was not depressed [PhQ-9 (m) = 6.33] and we found no change, *t* (29) = −0.469, *p* = 0.321. Even though a retrospective measure, we captured the PSS pre and post VR use and found no change, *t* (29) = −1.031, *p* = 0.155. Finally, due to interest in potential mechanisms of effect of VR, we also measured cognitive fusion (the ability to articulate and separate from one’s emotions) and saw no effect, *t* (20) = −0.117, *p* = 0.127. The descriptive statistics about our insignificant findings are included in Table 5.

## 4 LIMITATIONS

This was a small exploratory study with a mixed sample of adolescents, therefore generalization of our data are likely limited due to the following the small sample, age variability, and lack of controlled variables. We also do not know the effect of the pandemic on participant stress and study experience. As with any study with participant-reported outcomes, there is also a risk for social desirability bias. Finally, we were limited by the self-reporting of VR session activity. If a participant failed to complete a VR session log, we were unable to include their data in our analyses. Future studies will include VR session logs integrated into the VR system to avoid this limitation.

## 5 DISCUSSION

### 5.1 Use and Usability

Given the novelty of therapeutic virtual reality for adolescents, very little is known about experience of self-administering a virtual reality tool. Findings from this study indicate that adolescents were eager to use VR and, for the most part, enjoyed using the Nature Treks VR environment. The usability scores indicate Nature Treks VR had above average usability and enough engagement for repeated use for most participants. However, 10% of our participants indicated that they would not continue to use the Nature Treks environment due to disinterest or discomfort. Designing a VR environment that is continually engaging over a long period of time may be challenging for adolescents, especially given that they comprise a diverse population with unique and varied interests.

### 5.2 Emotional Experience

Although virtual reality is increasing in popularity as a therapeutic tool for anxiety-related problems, it is also heavily criticized for the limited research in children and adolescents (Kothgassner and Felnhofer, 2021). In a recent literature review by Baghaei et al. (2021) of virtual reality studies aimed to treat depression and anxiety, only two studies included adolescents. Each study had a very small sample size. In one study, virtual reality was combined with biofeedback and resulted in a decrease in anxiety in six of the eight participants, all of whom suffered from severe behavioral and psychiatric problems (Bossenbroek et al., 2020). The second study included four adolescents with Autism Spectrum Disorder and showed a self-reported reduction in anxiety during singing after using the VR environment (Adjorlu et al., 2019). It is plausible that VR nature environments, such as Nature Treks VR, could be a therapeutic tool to support emotional regulation, a key component of adolescent mental health. Emotional regulation, thought to precede problem solving, develops during adolescence (Ahmed et al., 2015) and supports adolescents in navigating their daily lives (Lennarz et al., 2019). In contrast, emotional dysregulation is associated with childhood trauma (Dvir et al., 2014) and strongly correlated with depression, suicidal ideation and many other psychological disorders (De Berardis et al., 2020). Most participants in our study did experience and perceive a relaxation effect and for some it was quite strong. Additionally, many teens described their VR experience as a nice break or escape. This is not surprising as VR, not unlike a video game or social break, may have benefits of allowing teens to temporarily exit stressful environments. The desire for escape is fairly strong during adolescence (Hides et al., 2019; Nordby et al., 2019).

### 5.3 Effect on Stress

Findings from this study reveal that participants experienced reductions in their momentary stress following VR use. Many described feeling more calm and that the environments provided an escape from stressors. This stress reduction effect was not reflected in the post-study stress measure, though, suggesting that the benefits of the Nature Treks VR environment may be temporary. Despite this, teens indicated interest in continuing to use VR to reduce stress if given the opportunity.

Although the research on the use of VR to reduce stress is minimal, some stress reducing effects have been found. Maarsingh et al. (2019) found a VR game combined with biofeedback was able to shift participants mindset about stress. They found a significantly more positive mindset in both healthy controls and mental healthy patients after playing the game. Tarrant et al. (2018) showed a reduction in stress in adults as measured by EEG during a nature-based 360° environment experienced in virtual reality. However, we have found no studies exploring the effect of VR on stress in adolescents, suggesting this is still a fairly novel, and much needed area of exploration.

## 6 CONCLUSION

Overall, findings from this study indicate that adolescents are eager to use VR and self-administration was feasible. While our study was a brief usability pilot, the reductions in momentary stress highlights the potential for VR as a tool for stress reduction interventions. This aligns with the emerging literature that VR is a favored platform for mental health interventions (Frewen et al., 2020). A logical next step will be to explore incorporating evidence-based mental health interventions, such as mindfulness, within VR. While mindfulness-based interventions have demonstrated some effects in teens (Patel et al., 2018; Lin et al., 2019), we anticipate the immersive experience of VR coupled with such interventions may enhance therapeutic effects. To ensure such an intervention is acceptable by teens, we recommend a human-centered design approach whereby teen end-users co-design such a future intervention. Presently, our team is immersed in such work and is eager to test our novel VR environment that incorporates mindfulness activities. We anticipate creative, scalable interventions such as ours will be needed to meet the exponential growth in teens requiring mental health services.

## Figures and Tables

**TABLE 1 |:** Summary characteristics of study cohorts.

Cohort	n	Time frame	Sample	Age (m)	Female	Male	Grade (m)

Cohort 1	9	spring 2019	University	18.89	7	2	13.44
Cohort 2	11	spring 2020^a^	University	18.78	5	6	13.67
Cohort 3	10	fall 2020^a^	High school	16.2	5	5	10.9
Total	30	–	–	17.97	17	13	12.7

a Study took place during the COVID-19 pandemic.

**TABLE 2 |:** Virtual reality usage metrics summary.

	Mean	SD	Min	Max

Frequency (sessions)	–	–	–	–
Cohort 1	5.00	1.66	1	6
Cohort 2	6.27	2.48	1	9
Cohort 3	6.5	2.59	3	10
All Cohorts	5.37	2.48	1	10

Duration (minutes)	–	–	–	–
Cohort 1	17.27	8.68	2	45
Cohort 2	20.47	17.74	6	110
Cohort 3	18.65	10.78	3	62
All Cohorts	18.77	12.76	2	110

**TABLE 3 |:** Findings of linear regression predicted the VR session-measures of momentary stress and mood.

Stress	Estimate	Std. Error	t value	*p* value

Intercept	51.755	1.735	29.825	< 0.001
Post session (Reference: pre-session)	−15.494	2.454	−6.314	< 0.001
*R-squared: 0.125*	–	–	–	–

Mood	Estimate	Std. Error	t value	*p* value

Intercept	50.001	1.543	32.4102	< 0.001
Post session (Reference: pre-session)	10.650	2.186	4.873	< 0.001
*R-squared: 0.08*	–	–	–	–

**TABLE 4 |:** Findings of linear regression to predict the VR session-measures of stress and mood based on the duration of each session.

	Estimate	Std. Error	t value	*p* value

Intercept	−15.233 5	2.730 2	−5.580	< 0.001
Stress change	−0.013 9	0.120 3	−0.116	0.908
*R-squared: 0.013*	–	–	–	–

Intercept	11.562 34	2.759 05	4.191	< 0.001
Mood change	−0.063 97	0.120 65	−0.530	0.597
*R-squared: 0.002*	–	–	–	–

**TABLE 5 |:** Means and standard deviations for *pre* and *post* measures of perceived stress scale (PSS), patient health questionnaire (PHQ-9), cognitive fusion questionnaire (CFQ-7).

Type of measure	Cohorts	*n*	Pre-mean	Pre-sd	Post-mean	Post-sd

Perceived stress scale (PSS)	1–3	30	22.83	6.97	22.1	6.68
Patient health questionnaire (PHQ-9)	1–3	30	6.33	4.2	6.1	3.89
Cognitive fusion questionnaire (CFQ-7)	2–3	21	27.52	8.79	25.76	7.56

## Data Availability

The raw data supporting the conclusion of this article will be made available by the authors upon request, without undue reservation.
